# Predicting treatment response to vancomycin using bacterial DNA load as a pharmacodynamic marker in premature and very low birth weight neonates: A population PKPD study

**DOI:** 10.3389/fphar.2023.1104482

**Published:** 2023-02-16

**Authors:** Amadou Samb, Rimke De Kroon, Koos Dijkstra, Marre Van Den Brand, Martine Bos, Frank Van Den Dungen, Agnes Veldkamp, Bram Wilhelm, Timo R. De Haan, Yuma A. Bijleveld, Marceline Tutu Van Furth, Paul Savelkoul, Noortje Swart, Ron Mathot, Mirjam Van Weissenbruch

**Affiliations:** ^1^ Department of Pharmacy and Clinical Pharmacology, Amsterdam UMC location University of Amsterdam, Amsterdam, Netherlands; ^2^ Department of Neonatology, Amsterdam, Netherlands; ^3^ Department of Pharmacy and Clinical Pharmacology, Amsterdam UMC location Vrije Universiteit Amsterdam, Amsterdam, Netherlands; ^4^ Department of Medical Microbiology and Infection Control, Amsterdam University Medical Center, location VU Medical Center, Amsterdam, Netherlands; ^5^ InBiome BV, Amsterdam, Netherlands; ^6^ Department of Pediatric Infectious Diseases and Immunology, Emma Children’s Hospital, Amsterdam Institute for Infection and Immunity, Amsterdam, Netherlands; ^7^ Department of Medical Microbiology, NUTRIM School of Nutrition and Translational Research in Metabolism, Maastricht University Medical Centre, Maastricht, Netherlands

**Keywords:** neonatology, vancomycin, NONMEM, pharmacodynamics, coagulase-negative staphylococci, bacterial DNA, pharmacokinetics, TDM

## Abstract

**Background:** While positive blood cultures are the gold standard for late-onset sepsis (LOS) diagnosis in premature and very low birth weight (VLBW) newborns, these results can take days, and early markers of possible treatment efficacy are lacking. The objective of the present study was to investigate whether the response to vancomycin could be quantified using bacterial DNA loads (BDLs) determined by real-time quantitative polymerase chain reaction (RT-qPCR).

**Methods:** VLBW and premature neonates with suspected LOS were included in a prospective observational study. Serial blood samples were collected to measure BDL and vancomycin concentrations. BDLs were measured with RT-qPCR, whereas vancomycin concentrations were measured by LC-MS/MS. Population pharmacokinetic–pharmacodynamic modeling was performed with NONMEM.

**Results:** Twenty-eight patients with LOS treated with vancomycin were included. A one-compartment model with post-menstrual age (PMA) and weight as covariates was used to describe the time PK profile of vancomycin concentrations. In 16 of these patients, time profiles of BDL could be described with a pharmacodynamic turnover model. The relationship between vancomycin concentration and first-order BDL elimination was described with a linear-effect model. Slope *S* increased with increasing PMA. In 12 patients, no decrease in BDL over time was observed, which corresponded with clinical non-response.

**Discussion:** BDLs determined through RT-qPCR were adequately described with the developed population PKPD model, and treatment response to vancomycin using BDL in LOS can be assessed as early as 8 h after treatment initiation.

## 1 Introduction

Late-onset sepsis (LOS) or nosocomial sepsis is a severe infectious neonatal condition characterized by bacteremia and a systemic inflammatory response that clinically manifests after the first 72 h of life and typically originates from indwelling central lines or catheters during hospital admission (Shane et al., 2017). Premature and/or very low birth weight (VLBW) neonates are at an increased risk for LOS due to an immature immune system and frequent exposure to invasive procedures in neonatal intensive care units (NICUs) (Stoll et al., 2004). Coagulase-negative staphylococci (CoNS) are the predominant pathogen in LOS (Dong and Speer, 2015). Approximately 21% of VLBW infants experience at least one episode of culture-proven LOS (Stoll et al., 2002). LOS has a high morbidity and mortality rate in premature and/or small for gestational age infants (Stoll et al., 2004). In cases of (suspected) LOS, the current standard of care is initiating treatment with broad-spectrum antibiotics (an aminoglycoside and a penicillin derivative) until the pathogen is identified by blood culture, which can take up to 48 h. Thereafter, narrow-spectrum antibiotic treatment targeting the isolated pathogen is initiated.

Intravenous (i.v.) vancomycin is given for 7 days in neonates with CoNS-positive LOS. Standardized loading doses of vancomycin are initiated, and subsequent doses may be corrected and individualized by means of therapeutic drug monitoring (TDM). However, the bactericidal efficacy of vancomycin is currently not assessed by readily accessible or reliable pharmacodynamic (PD) markers. Current vancomycin dosing regimens rely on indexed pharmacokinetic (PK) efficacy targets, based on the ratio of area under the curve (AUC_0–24h_) and minimal-inhibitory concentration (MIC) (Pacifici and Allegaert, 2012; Abdulla et al., 2021; Lee et al., 2021). As these PK/PD indices rely heavily on the MIC, these may be inadequate as MICs are highly variable between patients, bacterial strains, and over time (Rathi et al., 2016).

PD markers that accurately describe the early and gradual bactericidal action of vancomycin in the case of CoNS-positive LOS are needed. Bacterial DNA load (BDL), determined through real-time quantitative PCR (RT-qPCR), is a marker used for this purpose. The method has a short turnover time, is sensitive, biologically accurate, and most of all, requires a small volume of blood (Van den Brand et al., 2014; van den Brand et al., 2018). The objective of this study was to assess changes in BDL to characterize the early response to vancomycin treatment in CoNS-positive LOS. A population-PK/PD model was developed for quantifying the effect of vancomycin on BDL in CoNS-positive LOS. In addition, potential covariates that influence the measure of the bactericidal effect were explored. Using this model, we aimed to gain insight into the vancomycin bactericidal effect over time and evaluate PD and clinical responses within the first hours of vancomycin treatment. A quantified vancomycin effect on BDL could support the accuracy of TDM.

## 2 Materials and methods

### 2.1 Study design

This was a single-center, prospective, observational study during a consisting intervention in the Amsterdam University Medical Center (Division of Neonatology, Pediatrics Department, VU Medical Center, Amsterdam, The Netherlands). Serial blood samples were collected for determining antibiotic concentrations, BDLs, and inflammatory markers. The study was approved by the local ethics committee (METC Vrije Universiteit Medisch Centrum, Amsterdam, The Netherlands) prior to the inclusion of patients and recorded in the Dutch CCMO registry (file number: NL22434.029.08).

### 2.2 Study population

Inclusion of subjects took place between 1 February 2009 and 13 November 2014. Neonates with suspected LOS and/or meningitis who were either premature or VLBW were eligible for inclusion. For the identification of LOS, the definitions by van der Zwet et al. (2005) were adhered. Prematurity was defined as a gestational age (GA) <32 weeks, and VLBW was defined as a birth weight below 1,500 g. Patients with syndromal or chromosomal abnormalities and congenital metabolic disease were excluded from the study. Oral and written informed consent was a prerequisite for study participation. Following clinical practice at that time, the first-in-line treatment for LOS was intravenous amikacin (12 mg/kg/day) combined with intravenous benzylpenicillin (200,000 IU/kg/day). After the initial blood culture results were known, treatment was switched to a targeted antibiotic. For the research, only the studied patients with culture-proven CoNS infection and vancomycin treatment (20–48 mg/kg/day) in 1-hourly intravenous infusions were included in the analysis.

### 2.3 Sample collection

Whole blood samples were collected from newly inserted peripheral venous cannulas and (if available) from the central (umbilical) venous catheters. Blood samples (0.2 mL) for BDL measurement were collected at t = 0 h, t = 4 h, t = 24 h, and t = 48 h. The samples at t = 0 h were collected before the first vancomycin dose. Plasma samples (0.1 mL) were collected at t = 1 h, t = 2 h, t = 4 h, and t = 12 h for measuring vancomycin concentrations. After collection, the samples were stored at −20°C until analysis.

Additional clinical and anthropomorphic data such as dose information, GA, post-menstrual age (PMA), postnatal age (PNA), birth weight (BW), current weight (WT), length (LT), concomitant medication, C-reactive protein (CRP), and serum creatinine (SCr) were collected from the patients’ electronic medical files.

### 2.4 Real-time quantitative PCR

The design, validation, and evaluation of the used RT-qPCR method have been described in earlier publications (Van den Brand et al., 2014; van den Brand et al., 2018). In brief, 200 µL of EDTA anticoagulated whole blood samples was treated with TTE (1% Triton X-100, 20 mM Tris-HCl pH 8.3, 1 mM EDTA) twice for hemolysis and removal of hemoglobin. Next, the samples were incubated for 10 min in 200 µL of bacterial lysis buffer (Biocartis, Mechelen, Belgium) at 95°C while shaking at 800 rpm. Then, 20 µL of neutralization buffer (Biocartis, Mechelen, Belgium) was added to the solution, and DNA was purified using the NucliSENS EasyMag device (bioMérieux, Zaltbommel, The Netherlands). Samples were spiked with Phocine herpesvirus 1 as an internal control.

PCR was performed on a LightCycler 480II device (Roche Diagnostics, Almere, The Netherlands). 2 × LightCycler 490 Probes Master (12.5 µL), 2.5 µL primers and probes, and 10 µL purified DNA sample were used as reaction mixtures. The samples were screened for the eight most common pathogens of LOS in a multiplex assay (Van den Brand et al., 2014). Cycling conditions were 10 min at 95°C, followed by 45 cycles of 15 s at 95°C and 1 min at 60°C. If amplification was detected, the sample was then evaluated in a monoplex assay for quantitative analysis. BDLs were determined using a standard curve of serial dilutions of cloned PCR amplicons. The BDL was expressed in colony-forming units equivalent per ml (CFU eq/mL) by correcting for blood volumes and the number of PCR target copies per genome. The lower limit of quantification (LLOQ) for CoNS was 55 CFU eq/mL.

### 2.5 Liquid chromatography coupled to tandem mass spectrometry (LC-MS/MS)

Vancomycin concentrations were measured in plasma samples using LC-MS/MS. In brief, an Acquity TQD tandem quadrupole UPLC/MS/MS system (Waters, Milford, United States) was used, and the method had a within-run accuracy and an imprecision of at least 94.5% and at most 5.3%, respectively. Between-run accuracy and imprecision were at least 104.5% and at most 8.7%, respectively. The LLOQ of the used method was 0.22 mg/L (Chahbouni et al., 2015).

### 2.6 Bactericidal responses and clinical record evaluation

Changes in BDL over time following the initial vancomycin dose were plotted for all individuals in the study population. Thereafter, clinical records and treatment response patterns of patients were evaluated by a neonatologist of our research team for correspondence to BDL profiles.

### 2.7 Pharmacokinetic–pharmacodynamic data analysis

The BDL responses were modeled for all patients demonstrating a bactericidal response. Non-linear mixed-effects population PKPD (pop-PKPD) modeling was performed to describe and evaluate the relationship between vancomycin dose, concentration, and BDL and used to estimate pop-PKPD parameters. The model was developed using NONMEM version 7.4.0 software (Icon Development Solutions, Ellicott City, MD, United States). All data handling, data visualization, and descriptive statistics were performed using R statistics version 4.1.0 (R Core Team, 2021). Model validation and evaluation steps were performed with PsN version 5.3.0.

In a pop-PKPD analysis, data from all patients are analyzed simultaneously, allowing the analysis of patients for which only sparse samples are available. With sparse sampling, individual parameter estimates may be obtained by *post hoc* (Bayesian) analysis. Both “fixed effects” (typical parameters) and “random effects” (inter-individual variability (IIV) and residual variability) are estimated in pop-PKPD modeling. Thus, parameters such as clearance (CL) and the IIV in CL are embedded in the model. Part of the IIV may be explained by including covariates such as age in the model. Any remaining inaccuracies in predictions are included in the model as residual error. Parameter estimations were evaluated by assessing the objective function value (OFV), which is a maximum likelihood estimation-based approach. For parameter inclusion in nested models, a change in OFV of −3.84 corresponds with a *p*-value = 0.05 given 1° of freedom, which was deemed significant for parameter inclusion. After each modeling step, model accuracy was assessed by evaluating goodness-of-fit (GOF) plots, parameter relative standard error (RSE), and changes in OFV. Models were evaluated and internally validated using visual predictive check (VPC, *N* = 1,000) or prediction-corrected VPC (pc-VPC, *N* = 1,000), a simulation-based diagnostic to evaluate the predictive performance of the model. Model robustness and parameter certainty were assessed by a sampling importance resampling (SIR) procedure (Dosne et al., 2016; Dosne et al., 2017). Using the covariance matrix as the initial proposal distribution, five iterations with 1,000, 1,000, 1,000, 2,000, and 2,000 samples (M) and 200, 400, 500, 1,000, and 1,000 resamples (m) were performed during SIR.

The integrated PKPD model was developed using a sequential estimation approach. First, a vancomycin PK model found in literature was used for data description. Seven different published PK models were screened, selected on model evaluation and validation and similarities in the study population (Seay et al., 1994; Grimsley and Thomson, 1999; Capparelli et al., 2001; Kimura et al., 2004; Anderson et al., 2007; Marqués-Miñana et al., 2010; Oudin et al., 2011). The model with the highest precision, defined as the root mean squared error (*rmse*), was selected. It was a prerequisite for unbiased model performance, which was assessed using the distribution of the mean error (*me*). This was further supported by evaluation of GOF and VPC. Finally, the model that most accurately described the data was used as the foundation for the final model and used to reliably estimate the individual CL and volumes of distribution (V_d_) of the study population using the POSTHOC setting in NONMEM.

An empirical turnover PD model was appended to the PK model (Figure 2). In this PD model, it was assumed that there is both bacterial growth and bacterial decay in the absence of vancomycin, and the rates were parameterized as *k*_*growth*_ and *k*_*death*,_ respectively. Effect *E* of vancomycin was modeled to augment the effect of *k*_*death*_ in the model, as shown in differential Eq. 1.

$$\frac{\delta N}{\delta t}=k_{growth}*N-E*k_{death}*N.$$ (1)

where, *k*_*growth*_ is the first-order multiplication rate h^−1^, *k*_*death*_ is the first-order decay rate of in h^−1^, *N* is the BDL in CFU eq/mL, and *E* is the stimulatory effect of vancomycin on *k*_*death*_. If simultaneous estimation of k_growth_, k_death_, and effect parameters was not possible due to insufficient data, *k*_*growth*_ was defined as a zero-order constant (Eq. 2), changing the PD model to Eq. 3:

$$k_{growth}=\frac{BDL_{0}}{k_{death}}.$$ (2)

*k*_*growth*_ is the zero-order multiplication rate of BDL in CFU eq*ml^−1^*h^−1^, *k*_*death*_ is the first-order decay rate in h^−1^, and *BDL*_*0*_ is the estimated BDL in CFU eq/mL at t = 0 h.

$$\frac{\delta N}{\delta t}=k_{growth}-E*k_{death}*N.$$ (3)

Effect *E* was parameterized as either a linear-effect model (Eq. 4) or a sigmoidal *E*_*max*_ model (Eq. 5):

E=1+S*C. (4)

$$E=1+\frac{E_{\max}*C^{\gamma }}{EC_{50}^{\gamma }+C^{\gamma }}$$. (5)

In Eq. 4, Effect *E* is the product of the vancomycin concentration *C* and slope *S.* For the sigmoidal *E*_*max*_ model (Eq. 5), Effect *E* is described by maximum effect *E*_*max*_, vancomycin concentration *C*, the vancomycin concentration where 50% of the maximum effect is achieved (*EC*_*50*_), and a hill constant *γ* that describes the steepness of the effect.

Stepwise covariate analysis was performed, with a significance threshold of *p* = 0.05 for forward inclusion and *p* = 0.01 for backward elimination. GA, PMA, PNA, WT, BW, baseline, and CRP were evaluated as potential covariates. Continuous covariates were modeled as a linear or power function (Eqs 6, 7).

$$P=\theta_{p}*\left(1+\theta_{cov}*\frac{COV-COV_{median}}{COV_{median}}\right),$$ (6)

$$P=\theta_{p}*{\left(\frac{COV}{COV_{median}}\right)}^{\theta_{cov}},$$ (7)

where parameter *P* is expressed by typical parameter θ_p_, which is affected by deviation of covariate *COV* from the median covariate value *COV*_*median*_ with an effect of magnitude *θ*_*cov*_.

## 3 Results

### 3.1 Demographic characteristics

A total of 28 patients with CoNS-positive LOS and vancomycin treatment were included for analysis. The demographic characteristics of these patients are depicted in Table 1. A total of 94 vancomycin concentrations and 132 BDLs were available for analysis.

**TABLE 1:** Demographic characteristics of the study population.

Characteristic	
*Patients—N*	28
*Vancomycin samples—N*	94
*BDL samples—N*	132
*Female—N (%)*	9 (32.1%)
*GA (weeks)—median (IQR)*	28.4 (26.7–29.9)
*PNA (days)—median (IQR)*	11 (8–14)
*PMA (days)—median (IQR)*	214 (200–220)
*WT (grams)—median (IQR)*	1,110 (955–1,279)
*BW (grams)—median (IQR)*	1,150 (971–1,235)
*LT (cm)—median (IQR)*	37.5 (35.0–39.6)
*BLT (cm)—median (IQR)*	38.0 (35.0–39.1)
*SCr (µmol/L)—median (IQR)*	49.5 (44.8–55.3)

GA, gestational age; PNA, postnatal age; PMA, post-menstrual age; WT, bodyweight at inclusion; BW, birth weight; LT, length at inclusion; BLT, birth length; SCr, serum creatinine.

### 3.2 Bacterial DNA load response

**FIGURE 1:**
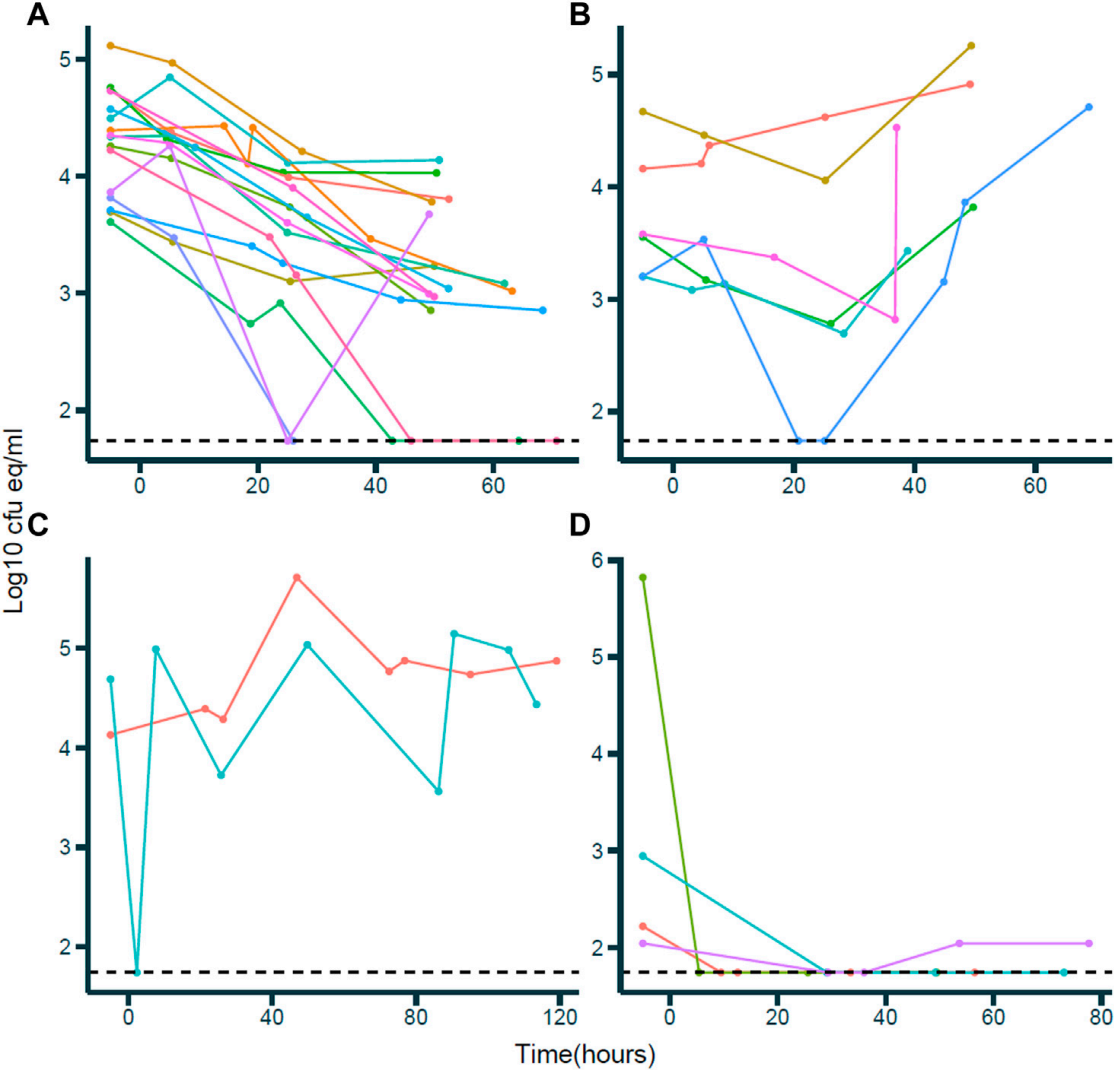
Bacterial DNA load (BDL)-time profiles during vancomycin treatment. *X*-axis: time in hours; *Y*-axis: BDL in log10 cfu eq/mL. Dots and lines of the same color within a plot indicate observations for individual patients. **(A)** Bactericidal responses; individuals demonstrating a negative trend in BDL over time. **(B)** Persisting septic responses; individuals demonstrating a constant or increasing trend in BDL over time. **(C)** Erratic responses; individuals demonstrating an erratic BDL over time following no specific pattern. **(D)** Unquantifiable response; individuals with very low BDL and BDL below quantification limit since vancomycin treatment.

Pooled BDL responses during vancomycin treatment are depicted in Figure 1. A bactericidal BDL response, defined as a decrease in BDL in 48 h, was observed in 16 patients, combined with clinical efficacy (Figure 1A). Six patients demonstrated persisting septic BDL profiles (Figure 1B). Five of these patients had complicated central line infections in which lines were not removed during BDL measurements, which explains the lack of clinical and BDL response since clinical response was observed upon line removal. The two erratic profiles (Figure 1C) could be explained by patients having an infected thrombus and an infected line obstructed by a pustule, respectively. Four patients had near-unquantifiable BDL profiles (Figure 1D). However, in three of these patients, amikacin-susceptible CoNS were isolated and, therefore, cured due to the empirical pretreatment with amikacin. The persisting septic, erratic, and unquantifiable profiles were pooled as ‘non-response’ (*n* = 12).

### 3.3 Vancomycin pharmacokinetic–pharmacodynamic model

Seven published PK models for vancomycin in pediatric/neonatal populations were evaluated to fit the study data. The one-compartment model by Marqués-Miñana et al. (2010) (Table 2) was most accurate in describing the vancomycin levels of our study population. This was decided based on precision, as defined by the lowest *rmse* and bias, which was evaluated using the confidence interval of *me* (Sheiner and Beal, 1981) (Salimnia et al., 2016). An *rmse* of 2.959 mg/L was observed, which was the lowest of all tested models. The *me* was 0.147 mg/L (−0.453–0.747 mg/L), indicating unbiased model performance. This was supported by GOF plots and pc-VPC (Supplementary Figure S1). While the model included comedication with spironolactone and amoxicillin as covariates, no patients received any of these drugs during the study period. The PK model was used to estimate the individual CL and V_d_ of the study population.

**TABLE 2:** Structural model, final model, and SIR results for the plasma and saliva model.

	Structural model		Final model		SIR results (five iterations)	
	OFV = 81.361		OFV = 69.883		*M* = 1,000, 1,000, 1,000, 2000, and 2000 *m* = 200,400,500,1000, and 1,000	
Parameter	Estimate	RSE	Estimate	RSE	Mean estimate (95% CI)	RSE
Plasma model Marqués-Miñana et al. (2010)						
θCL (l* h^−1^ *kg^−1^ *wk^−1^)	0.00192	—	0.00192	—	—	—
θV (L kg^−1^)	0.572	—	0.572	—	—	—
θAMX-CL	0.65	—	0.65	—	—	—
θSPI-V	0.344	—	0.344	—	—	—
ωCL	35.6%	—	35.6%	—	—	—
ωV	19.3%	—	19.3%	—	—	—
σ_add_ (mg L^−1^)	2.69	—	2.69	—	—	—
Saliva model						
θ_kdeath_ (h^−1^)	0.0033	31%	0.0035	31%	0.0037 (0.0016–0.0065)	37%
θ_S_	0.862	36%	0.833	32%	0.968 (0.503–1.893)	37%
θ_PMA_	—	—	8.23	31%	7.76 (3.23–13.27)	33%
θ_BDL-0_	10,600	24%	10,400	22%	11,213 (7,815–15717)	18%
θ_BDL-S_	1.13	25%	1.02	20%	1.17 (0.974–1.327)	8%
ω_S_ (shrinkage)	64.1% (24%)	71%	58.8% (20%)	36%	45% (12.6%–72.5%)	40%
ω_BDL-s_ (shrinkage)	72.0% (11%)	18%	5.8% (11%)	27%	60% (34.6%–83.1%)	23%
σ_prop_ (shrinkage)	0.163 (27%)	16%	16.3% (27%)	16%	17.7% (13.0%–24.8%)	16%

θ_cl_, clearance; PMA, post-menstrual age; WT, bodyweight; AMX, amoxicillin comedication; θ_AMX-CL_, amoxicillin effect on clearance; θ_V_, volume of distribution; SPI, spironolactone use; θ_SPI-V_, spironolactone effect on volume of distribution; θ_kdeath_, first-order rate constant for natural bacterial death. θ_S_, slope of the linear-effect model; θ_PMA_, power equation exponent of PMA on θ_S_; θ_BDL0_, typical BDL_0_; θ_BDL0-S_, slope of linear BDL_0_ function; ωS, inter-individual variability in slope S; ω_BDL0-s_, inter-individual variability in typical BDL_0_; σ_prop_, proportional residual error; OFV, objective function value. The BDL_0_ was scaled with the median BDL_0_ of 14520.

**FIGURE 2:**
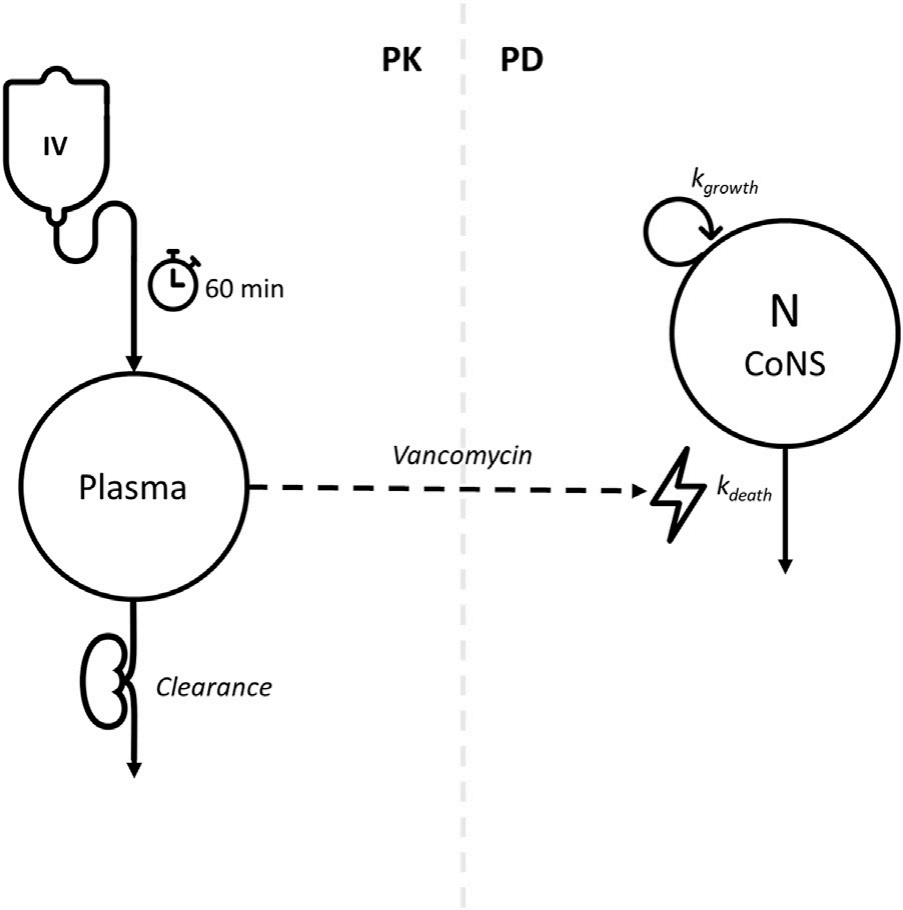
Conceptual pharmacokinetic–pharmacodynamic model for vancomycin effect on bacterial DNA load. Left side of the dashed line: one-compartment PK model. Vancomycin is infused intravenously for 1 h and is renally cleared from the body. Right side of the dashed line: empirical turnover PD model. The number of CoNS (N) has a natural *in vivo* multiplication rate of k_growth_ and natural cell death rate of k_death_. The vancomycin concentration in plasma has a bactericidal action by stimulation of k_death_, depicted by the dashed arrow connecting the left and right sections of the figure.

A population PK-PD model was developed using only the data of the patients demonstrating a bactericidal response to vancomycin (*n* = 16; Figure 1A). We attempted to develop a model including all patients, though no model could be fit due to data limitations. Pharmacodynamics was described with an empirical turnover model (Figure 2). Parameter estimates of the model are summarized in Table 2. The natural BDL decay was described in the structural model with the first-order rate constant *k*_*death*_ of 0.0033 h^−1^. The stimulatory effect *E* of vancomycin on *k*_*death*_ was expressed as a linear-effect model (Eq. 4), with a slope *S* of 0.862. The IIV of the slope was 64.1%. Due to limited data, the natural growth constant *k*_*growth*_ could not be estimated as an independent parameter. Therefore, *k*_*growth*_ was expressed as Eq. 2 and was a zero-order rate constant dependent on the initial BDL_0_ at t = 0 h and *k*_*death*_. BDL_0_ was estimated, and the last measured BDL before vancomycin treatment (BL) was included as a linear structural covariate. The IIV in BDL_0_ was 72% and was slightly correlated with the IIV of *S*, as indicated by an off-diagonal of 30.1% in the omega matrix. The proportional residual error was 16.3%.

**FIGURE 3:**
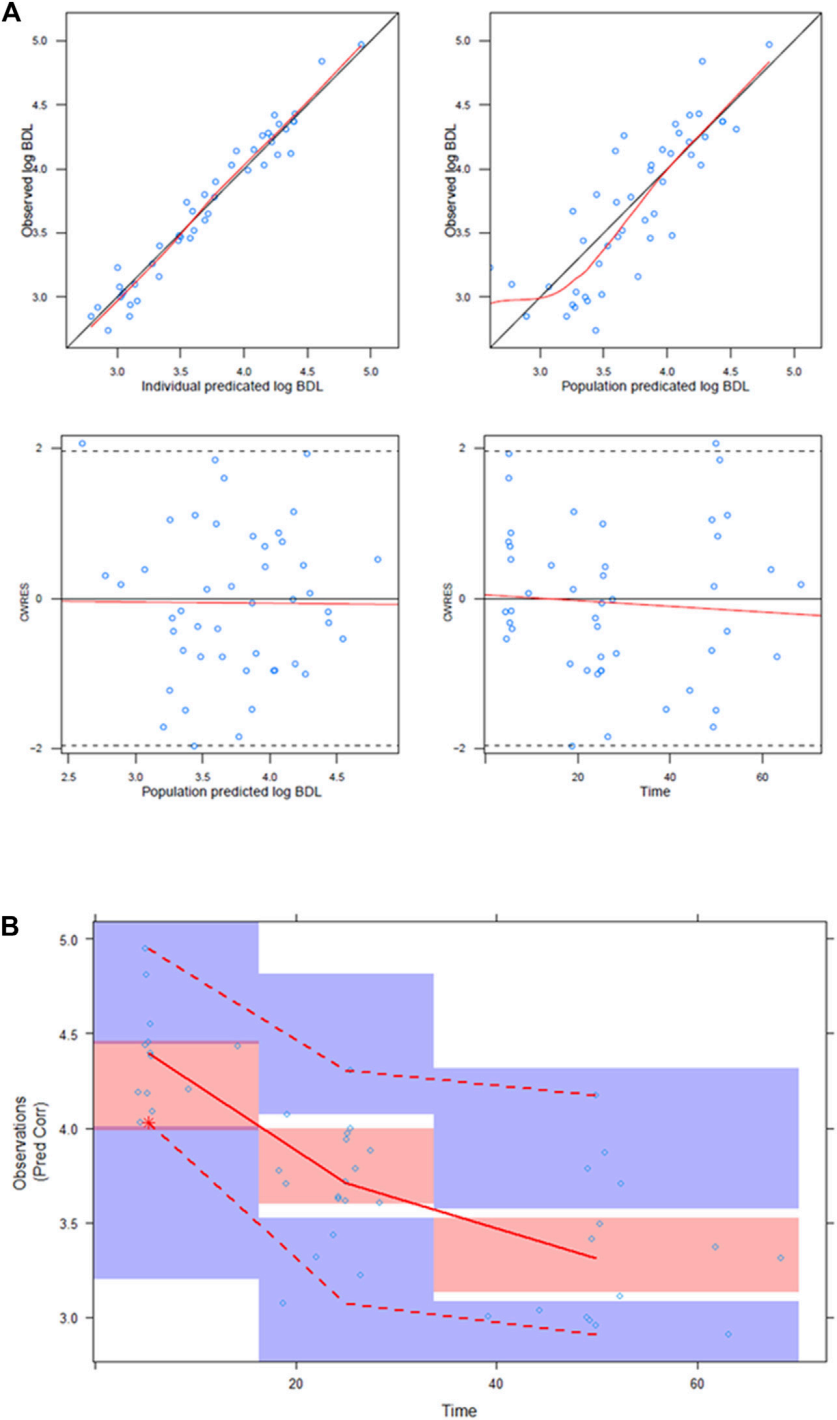
Goodness-of-fit plots and prediction-corrected VPC of the final model: **(A)** Goodness-of-fit plots for the PD model. Top left; individual log10 BDL predictions vs. log10 BDL observations. Top right; population log10 BDL predictions vs. log10 BDL observations. Bottom left; population BDL log10 predictions vs. CWRES. Bottom right; time after last dose vs. CWRES. CWRES: conditional weighted residuals. **(B)** Prediction-corrected VPC of the PD model (*n* = 1,000). *X*-axis: time after last dose; *Y*-axis, prediction-corrected log10 BDL. Black dots: observations. Blue lines: 10th and 90th percentiles of observations. Red line: median of observations. Blue shaded areas: 95% CIs of simulated 10th and 90th percentiles. Red shaded area: 95% CI of simulated median.

**FIGURE 4:**
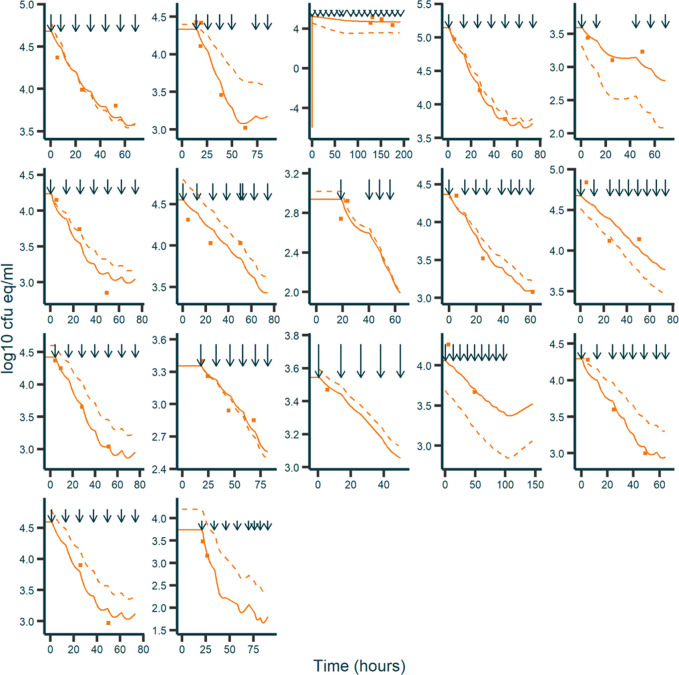
Fitted and observed BDL over time for individual responders. *X*-axis: time in hours. *Y*-axis: BDL in log10 cfu eq/mL. Dark blue arrows: vancomycin dosing times. Orange squares: observations. Solid orange line: individual predictions. Dashed orange line: population predictions.

In the covariate analysis, PMA was significantly associated with slope *S*. Using a power model (Eq. 7), an exponent of 8.2 was estimated, thereby decreasing the IIV of the S from 64.1% to 58.8% (Table 2). The correlation between the IIV in S and BDL in the off-diagonal of the omega matrix was increased to 72.3% for the final model. Relative standard errors of all parameters were within the acceptable range. GOF plots of the final model indicated an adequate model fit to the data (Figure 3). The bactericidal effect of vancomycin on individual patients is depicted in Figure 4.

Model performance was evaluated using a pc-VPC (*N* = 1,000) (Figure 3). The upper, median, and lower percentiles of the observations were within the 95% CIs of the corresponding simulated percentiles, indicating that the model could adequately predict the BDL levels of patients responding to treatment as a function of vancomycin PK. To assess model robustness and parameter uncertainty, SIR was performed. The SIR results of the final model are shown in Table 2; Supplementary Figure S2. The proposed parameter distribution was above the reference distribution. After the final iteration, the dOFV plots showed a chi-squared distribution with few degrees of freedom than the number of parameters in the model. No temporal trends were observed, and therefore the SIR results were accepted. There was full coverage of the final parameters.

## 4 Discussion

In this study, bacterial DNA loads were used as a PD marker to evaluate the bactericidal effect of vancomycin in premature and/or VLBW neonates with CoNS-positive late-onset sepsis. In patients demonstrating a bactericidal response, the time profile of the BDL could be quantified with an empirical PK/PD model. Using this model, the time course and measure of the bactericidal effect of vancomycin could be described (Figure 4).

To our knowledge, the present study is the first to quantify the bactericidal effect of vancomycin in premature and VLBW neonates with CoNS-positive LOS by assessing time profiles of BDL using RT-qPCR. Bacterial PCR has been investigated in the past as a potential diagnostic tool and for antimicrobial susceptibility screening in the context of LOS, and varying results have been reported, with some studies suggesting that BDL measured through PCR methods could serve as a surrogate for blood culture in diagnosing some infectious diseases (Van den Brand et al., 2014; Chahbouni et al., 2015), while others demonstrated that PCR-based diagnostics were inferior to conventional blood culture (Tröger et al., 2016; Morrissey et al., 2017). All studies indicate that PCR-based diagnostics are feasible, however.

After assessing previously published population PK models for vancomycin, the model by Marqués-Miñana et al. (2010) was found to best describe the observed vancomycin levels of our study population (Seay et al., 1994; Grimsley and Thomson, 1999; Capparelli et al., 2001; Kimura et al., 2004; Anderson et al., 2007, and; Oudin et al., 2011). The selected model was developed using data from 70 neonates admitted to an NICU and treated with vancomycin, with a large proportion of patients comparable to our study population (Marqués-Miñana et al., 2010). The model was internally and externally validated by the original authors.

For patients responding to vancomycin treatment, time profiles of BDL were described using a turnover model (Figure 2). There was a positive relation between slope *S* and PMA when PMA was included as a power function with an estimated exponent of 8.23, indicating that older patients demonstrated a larger bactericidal effect than younger patients at equal vancomycin concentrations. However, it is questionable whether this perceived age dependency in the bactericidal activity of vancomycin is truly the underlying process for the increased BDL decline for patients of higher PMA. Innate and adaptive immunity is immature for premature neonates, and it is possible that increased bactericidal action at higher PMA is a product of higher immune activity, rather than an increased effect of vancomycin (Collins et al., 2018). However, the data did not support the estimation of IIV on *k*_*death*_, nor could PMA be estimated as a covariate on *k*_*death*_. Nonetheless, including PMA in the model as an exponential function on slope *S* significantly improved model fit and explained 5.3% of the IIV in slope *S*. Still, the remaining IIV in slope *S* in the final model was 58.8% and could be considered very high for classical PKPD models. This high variability is likely the result of oversimplification of the model. A true mechanistic antimicrobial PKPD model incorporates a complicated system of true bacterial growth, decay, and sigmoidal E_*max*_ effects, as well as resistance mechanisms, ideally with multiple levels of IIV. Oversimplification of these underlying mechanisms results in a model where all these types of variability have been combined in a single, large IIV for slope. As for high IIV in BDL_0_, this is strongly supported by the data since the included patients demonstrated enormous variability in BDL at the first vancomycin dose.

A number of mechanistic PKPD models for antimicrobials have been published; however, these models have been exclusively applied in *in vitro* and animal studies. No such models could be found using clinical PD data. An overview of published antimicrobial PKPD models has been provided in a database by Minichmayr et al. (2022). A single similar publication was found that modeled the effect of vancomycin on CoNS colonization in central line-associated LOS using an *in vitro* hollow fiber infection model and a rabbit model (Ramos-Martín et al., 2016). The authors found that based on a translational model using their preclinical data, currently accepted dosing guidelines of AUC/MIC ≥400 were potentially too low for neonates ≤29 weeks GA and argued that efforts should be made for developing more efficacious dosing regimens in central line-associated LOS, optimizing bactericidal efficacy, minimizing toxicity, and preventing drug resistance.

Since the analyzed population was small, bootstrapping methods were deemed unsuitable for evaluating parameter uncertainty and model stability (Dosne et al., 2017). Thus, SIR was used for this purpose. SIR converged after five iterations (Supplementary Figure S2), and reliable RSE and 95% confidence intervals were obtained (Table 2). Based on the SIR results, the model was deemed accurate.

Simulation-based model evaluation using pc-VPC (*N* = 1,000) indicated that the final model could adequately predict the observed BDLs in the study population. Therefore, the final constructed model was deemed suitable for this study and provided sufficient insight that the effect of vancomycin on BDLs as determined through RT-qPCR can be described in a population-PKPD model.

Six patients demonstrated an increasing or constant BDL, as demonstrated in Figure 1B. These “persisting septic” BDL responses were evaluated by a neonatologist in our research team using the recorded clinical response of the patient. Indeed, five of these patients had clinically persistent CoNS bacteremia during vancomycin therapy, where the primary source of LOS was CoNS colonization of the central venous line (CVL) *in situ,* and removal of the CVL resulted in clinical improvements and negative blood cultures. Although CVL infections are a frequent source of nosocomial sepsis, there seems to be little consensus on whether CVL removal is beneficial in CoNS bacteremia (Cairns et al., 1995; Benjamin et al., 2000; Benjamin et al., 2001). It has been found that in over 70% of CoNS CVL infections, line retention does not interfere with antimicrobial efficacy (Cairns et al., 1995). However, Benjamin et al. (2001) stated that CVL removal should be considered for patients with persistent sepsis, identified as four consecutive blood cultures positive for CoNS. Two of our patients presented erratic BDL profile measurements, showing no specific pattern in BDL change over time (Figure 1C). In one of these two patients, CoNS bacteremia was secondary to an infected thrombus. Presence of an infected thrombus has been implied as a risk factor for persistent or recurrent staphylococcal sepsis in multiple case reports (Hubbard et al., 2016; Mani and Chandrasekharan, 2022). Incremental degradation of the colonized thrombus due to shearing stress and releasing CoNS-infected debris into the bloodstream at irregular time intervals could explain the observed erratic BDL profile. The other patient suffered from an infected peripheral venous line, complicated by the presence of a pustule at the ankle. Likewise, irregular mechanical stress at the primary site of infection could release high loads of infected material into the bloodstream at random intervals. For some patients, most measured BDLs were below or near the quantification limit following vancomycin treatment (Figure 1D). In these cases, the isolated CoNS culture was susceptible to amikacin. During this study, empirical amikacin and benzylpenicillin treatment was a clinical routine until diagnostic blood culture results. Therefore, susceptible CoNS exposed to 48 h of amikacin is expected to demonstrate substantial bacterial killing, explaining the absence of CoNS BDL during subsequent vancomycin therapy.

There were some limitations to this study. First, since RT-qPCR quantifies the total bacterial DNA in the study sample, it could not distinguish between DNA from living or dead bacteria. While it is known that circulating free DNA has a half-life of 1–2 h and is cleared through macrophage-mediated phagocytosis and enzymatic degradation in the spleen and liver, to our best knowledge, the rate at which dead bacteria are cleared from the neonatal bloodstream is unknown (Kustanovich et al., 2019). To account for the time delay between vancomycin dosing and BDL decrease in the model, we attempted to estimate the lag time (T_lag_). However, including a T_lag_ in the model did not increase model fit and overcomplicated the model. Using total BDL allowed for a comprehensive approximation of bactericidal activity. Another limitation of the study was the risk of sample contamination. CoNS are not only the most frequent pathogen in LOS in developed countries but also the predominant contaminating micro-organism in blood samples (Huebner and Goldmann, 1999). Therefore, efforts should be made to minimalize the contamination risk. For instance, assessment of bacterial density could be considered or multiple sample sources could be used (Kassis et al., 2009). This was not performed during this study, as this would result in an unacceptable burden due to increased blood sampling. Regardless of this, culture-based assessments should be combined with careful clinical examination of patients to minimize the risk of unnecessary treatment due to sample contamination. The final limitation of the study is that no BDL profiles in the absence of antibiotic treatment were available. Therefore, it was difficult to distinguish between natural bacterial growth and treatment and thus estimate *k*_*growth*_ *, k*_*death*_ *,* and slope *S* as separate parameters. Unfortunately, no values of *k*_*growth*_ *or k*_*death*_ of CoNS, either as initial estimates or fixed parameters, could be found in the literature. A single study was identified that investigated the mechanistic PKPD relations between vancomycin and CoNS based on *in vitro* and animal data, though no parameter estimates were published in the model (Ramos-Martín et al., 2016). In our final model, it was assumed that *k*_*growth*_ was a zero-order constant dependent on BDL at *T* = 0 and *k*_*death*_ *.* As cellular multiplication relies on cell doubling, it is most certainly a first-order process. Moreover, relating *k*_*growth*_ to BDL at *T* = 0 results in a function in which the BDL cannot exceed this value at the cost of model accuracy. By estimating the BDL_0_ with IIV, the model could more accurately predict BDLs above the last BDL before the first vancomycin dose.

There were a number of strengths to this study and its implications. First, an empirical model was developed to describe the bactericidal action of vancomycin in CoNS-positive LOS. CoNS are the predominant infective pathogen in LOS, accounting for approximately 53.2%–77.9% of all culture-proven LOS cases in developed countries (Dong and Speer, 2015). Vancomycin is the first-in-line antibiotic in CoNS-positive LOS, and dosing guidelines in neonatology are currently based on an AUC_24h_/MIC index, in which a target of at least 400 is generally associated with efficacy (Pacifici and Allegaert, 2012). However, PK/PD indices heavily rely on the MIC, which is associated with considerable variability between bacterial strains, patients, and occasions (Rathi et al., 2016). Moreover, these indices treat bactericidal action as a binary “all-or-nothing” response. This implies that bacterial killing is only active at concentrations above the MIC and inactive at levels below the MIC, whereas in reality, bacterial killing changes dynamically with concentration. The method proposed here incorporates gradual bacterial killing as a function of vancomycin concentration and provides a more nuanced insight into bactericidal dynamics, independent of the MIC. This could be of particular benefit in the context of TDM, as improved concentration targets could be identified. Second, a relatively large number of drug concentrations and BDLs were available for each enrolled patient. Blood sampling in neonatology comes with considerable risk, and the number of samples collected per patient is hampered in the research context (Howie, 2011). Therefore, studies with a large amount of samples per patient are infrequent and valuable in this population. The relatively large amount of measurements per patient in our study allowed for more accurate depictions of the underlying PK and PD principles. A third strength of this study was that bacterial blood colonization was determined through multiplex RT-qPCR. The method used was validated and evaluated in the clinical setting and allowed for quantification of CFU eq/mL by adjusting measured DNA load for sample volume and CoNS genome load (Van den Brand et al., 2014; van den Brand et al., 2018). Thus, a surrogate marker for blood colonization that could be quantified within 8 h provided information on the bactericidal action of vancomycin in this study. Last, BDL profiles indicating treatment non-response (Figures 1BD) were compared with the corresponding clinical records by a neonatologist to assess whether these patients did not respond clinically. By doing so, the assumption to create the PKPD model based only on data of patients demonstrating a decrease in BDL was confirmed.

This study demonstrates that a decrease in BDL in CoNS-positive LOS can be quantified and predicted as a function of vancomycin concentration over time for patients who respond to vancomycin therapy. If developed further by combining preclinical data with clinical data, this would allow for more nuanced and precise dosing regimens, as compared to the currently used “all-or-nothing” dosing guidelines based on MIC targets. Moreover, it is expected that the applicability and accuracy of TDM could significantly improve if more evidence-based targets are identified.

## Data Availability

The raw data supporting the conclusions of this article will be made available by the authors, without undue reservation.

## References

[B1] Abdulla A. Edwina A. E. Flint R. B. Allegaert K. Wildschut E. D. Koch B. C. P. (2021). Model-informed precision dosing of antibiotics in pediatric patients: A narrative review. Front. Pediatr. 1–11, 624639. 10.3389/fped.2021.624639 PMC7940353 33708753

[B2] Anderson B. J. Allegaert K. Van Den Anker J. N. Cossey V. Holford N. H. G. (2007). Vancomycin pharmacokinetics in preterm neonates and the prediction of adult clearance. Br. J. Clin. Pharmacol. 63 (1), 75–84. 10.1111/j.1365-2125.2006.02725.x 16869817 PMC2000709

[B3] Benjamin D. K. Miller W. Garges H. Benjamin D. K. McKinney R. E. Cotton M. (2001). Bacteremia, central catheters, and neonates: When to pull the line. Pediatrics 107 (6), 1272–1276. 10.1542/peds.107.6.1272 11389242

[B4] Benjamin D. K. Ross K. McKinney R. E. Benjamin D. K. Auten R. Fisher R. G. (2000). When to suspect fungal infection in neonates: A clinical comparison of Candida albicans and Candida parapsilosis fungemia with coagulase-negative staphylococcal bacteremia. Pediatrics 106 (4), 712–718. 10.1542/peds.106.4.712 11015513

[B5] Cairns P. A. Wilson D. C. McClure B. G. Halliday H. L. McReid M. (1995). Percutaneous central venous catheter use in the very low birth weight neonate. Eur. J. Pediatr. 154 (2), 145–147. 10.1007/BF01991919 7720744

[B6] Capparelli E. V. Lane J. R. Romanowski G. L. McFeely E. J. Murray W. Sousa P. (2001). The influences of renal function and maturation on vancomycin elimination in newborns and infants. J. Clin. Pharmacol. 41 (9), 927–934. 10.1177/00912700122010898 11549096

[B7] Chahbouni A. Van Den Dungen F. A. M. Vos R. M. Den Burger J. C. G. Sinjewel A. Wilhelm A. J. (2015). An UPLC-MS detection method for the quantification of five antibiotics in human plasma. Bioanalysis 7 (18), 2321–2329. 10.4155/bio.15.121 26417882

[B8] Collins A. Weitkamp J. H. Wynn J. L. (2018). Why are preterm newborns at increased risk of infection? Arch. Dis. Child. Fetal Neonatal Ed. 103 (4), F391–F394. 10.1136/archdischild-2017-313595 29382648 PMC6013388

[B9] Dong Y. Speer C. P. (2015). Late-onset neonatal sepsis:Recent developments. Arch. Dis. Child. Fetal Neonatal Ed. 100 (3), F257–F263. 10.1136/archdischild-2014-306213 25425653 PMC4413803

[B10] Dosne A. G. Bergstrand M. Harling K. Karlsson M. O. (2016). Improving the estimation of parameter uncertainty distributions in nonlinear mixed effects models using sampling importance resampling. J. Pharmacokinet. Pharmacodyn. 43 (6), 583–596. 10.1007/s10928-016-9487-8 27730482 PMC5110709

[B11] Dosne A. G. Bergstrand M. Karlsson M. O. (2017). An automated sampling importance resampling procedure for estimating parameter uncertainty. J. Pharmacokinet. Pharmacodyn. 44 (6), 509–520. 10.1007/s10928-017-9542-0 28887735 PMC5686280

[B12] Grimsley C. Thomson A. H. (1999). Pharmacokinetics and dose requirements of vancomycin in neonates. Arch. Dis. Child. - Fetal Neonatal Ed. 81 (3), F221–F227. 10.1136/fn.81.3.f221 10525029 PMC1721000

[B13] Howie S. R. (2011). Blood sample volumes in child health research: Review of safe limits. Bull. World Health Organ 89 (1), 46–53. 10.2471/BLT.10.080010 21346890 PMC3040020

[B14] Hubbard E. Wise E. Hubbard B. Girard S. Kong B. Moudgal V. (2016). Tucked away: An infected thrombus. Am. J. Med. 129 (6), 576–579. 10.1016/j.amjmed.2016.01.042 26901112

[B15] Huebner J. Goldmann D. A. (1999). Coagulase-negative staphylococci: Role as pathogens. Annu. Rev. Med. 50, 223–236. 10.1146/annurev.med.50.1.223 10073274

[B16] Kassis C. Rangaraj G. Jiang Y. Hachem R. Y. Raad I. (2009). Differentiating culture samples representing coagulase-negative staphylococcal bacteremia from those representing contamination by use of time-to-positivity and quantitative blood culture methods. J. Clin. Microbiol. 47 (10), 3255–3260. 10.1128/JCM.01045-09 19692564 PMC2756952

[B17] Kimura T. Sunakawa K. Matsuura N. Kubo H. Shimada S. Yago K. (2004). Population pharmacokinetics of arbekacin, vancomycin, and panipenem in neonates. Antimicrob. Agents Chemother. 48 (4), 1159–1167. 10.1128/aac.48.4.1159-1167.2004 15047516 PMC375245

[B18] Kustanovich A. Schwartz R. Peretz T. Grinshpun A. (2019). Life and death of circulating cell-free DNA. Cancer Biol. Ther. 20 (8), 1057–1067. 10.1080/15384047.2019.1598759 30990132 PMC6606043

[B19] Lee S. M. Yang S. Kang S. Chang M. J. (2021). Population pharmacokinetics and dose optimization of vancomycin in neonates. Sci. Rep. 11 (1), 6168–8. 10.1038/s41598-021-85529-3 33731764 PMC7969932

[B20] Mani S. Chandrasekharan P. (2022). Staphylococcus lugdunensis bacteremia with an infected aortic thrombus in a preterm infant. Children 9 (1), 46–48. 10.3390/children9010046 35053671 PMC8774124

[B21] Marqués-Miñana M-R. Saadeddin A. Peris J-E. (2010). Population pharmacokinetic analysis of vancomycin in neonates. A new proposal of initial dosage guideline. Br. J. Clin. Pharmacol. 70 (5), 713–720. 10.1111/j.1365-2125.2010.03736.x 21039765 PMC2997311

[B22] Minichmayr I. K. Aranzana-Climent V. Friberg L. E. (2022). Pharmacokinetic-pharmacodynamic models for time courses of antibiotic effects. Int. J. Antimicrob. Agents 60, 106616. 10.1016/j.ijantimicag.2022.106616 35691605

[B23] Morrissey S. M. Nielsen M. Ryan L. Al Dhanhani H. Meehan M. McDermott S. (2017). Group B streptococcal PCR testing in comparison to culture for diagnosis of late-onset bacteraemia and meningitis in infants aged 7–90 days: A multi-centre diagnostic accuracy study. Eur. J. Clin. Microbiol. Infect. Dis. 36 (7), 1317–1324. 10.1007/s10096-017-2938-3 28247153

[B24] Oudin C. Vialet R. Boulamery A. Martin C. Simon N. (2011). Vancomycin prescription in neonates and young infants: Toward a simplified dosage. Arch. Dis. Child. Fetal Neonatal Ed. 96 (5), 365–370. 10.1136/adc.2010.196402 21378399

[B25] Pacifici G. Allegaert K. (2012). Clinical pharmacokinetics of vancomycin in the neonate: A review. Clinics 67 (7), 831–837. 10.6061/clinics/2012(07)21 22892931 PMC3400177

[B26] R Core Team (2021). R A language and environment for statistical computing. Vienna, Austria: Springer.

[B27] Ramos-Martín V. Johnson A. Livermore J. McEntee L. Goodwin J. Whalley S. (2016). Pharmacodynamics of vancomycin for CoNS infection: Experimental basis for optimal use of vancomycin in neonates. J. Antimicrob. Chemother. 71 (4), 992–1002. 10.1093/jac/dkv451 26755499 PMC4790623

[B28] Rathi C. Lee R. E. Meibohm B. (2016). Translational PK/PD of anti-infective therapeutics. Drug Discov. Today Technol. 21–22, 41–49. 10.1016/j.ddtec.2016.08.004 PMC5172400 27978987

[B29] Salimnia H. Fairfax M. R. Lephart P. R. Schreckenberger P. DesJarlais S. M. Johnson J. K. (2016). Evaluation of the FilmArray blood culture identification panel: Results of a multicenter controlled trial. J. Clin. Microbiol. 54 (3), 687–698. 10.1128/JCM.01679-15 26739158 PMC4767991

[B30] Seay R. E. Brundage R. C. Jensen P. D. Schilling C. G. Edgren B. E. (1994). Population pharmacokinetics of vancomycin in neonates. Clin. Pharmacol. Ther. 56 (2), 169–175. 10.1038/clpt.1994.120 8062493

[B31] Shane A. L. Sánchez P. J. Stoll B. J. (2017). Neonatal sepsis. Lancet 390 (10104), 1770–1780. 10.1016/S0140-6736(17)31002-4 28434651

[B32] Sheiner L. B. Beal S. L. (1981). Some suggestions for measuring predictive performance. J. Pharmacokinet. Biopharm. 9 (4), 503–512. 10.1007/BF01060893 7310648

[B33] Stoll B. J. Hansen N. Fanaroff A. A. Wright L. L. Carlo W. A. Ehrenkranz R. A. (2002). Late-onset sepsis in very low birth weight neonates: The experience of the NICHD Neonatal Research Network. Pediatrics 110 (2), 285–291. 10.1542/peds.110.2.285 12165580

[B34] Stoll B. J. Hansen N. I. Adams-Chapman I. Fanaroff A. A. Hintz S. R. Vohr B. (2004). Neurodevelopmental and growth impairment among extremely low-birth-weight infants with neonatal infection. J. Am. Med. Assoc. 292 (19), 2357–2365. 10.1001/jama.292.19.2357 15547163

[B35] Tröger B. Härtel C. Buer J. Dördelmann M. Felderhoff-Müser U. Höhn T. (2016). Clinical relevance of pathogens detected by multiplex PCR in blood of very low birth weight infants with suspected sepsis – multicentre study of the German neonatal network. PLoS One 11 (7), 01598211–e159910. 10.1371/journal.pone.0159821 PMC4966931 27472282

[B36] Van den Brand M. Peters R. P. H. Catsburg A. Rubenjan A. Broeke F. J. Van den Dungen F. A. M. (2014). Development of a multiplex real-time PCR assay for the rapid diagnosis of neonatal late-onset sepsis. J. Microbiol. Methods 106, 8–15. 10.1016/j.mimet.2014.07.034 25102109

[B37] van den Brand M. van den Dungen F. A. M. Bos M. P. van Weissenbruch M. M. van Furth A. M. de Lange A. (2018). Evaluation of a real-time PCR assay for detection and quantification of bacterial DNA directly in blood of preterm neonates with suspected late-onset sepsis. Crit. Care 22 (1), 105–110. 10.1186/s13054-018-2010-4 29679983 PMC5911371

[B38] van der Zwet W. C. Kaiser A. M. van Elburg R. M. Berkhof J. Fetter W. P. F. Parlevliet G. A. (2005). Nosocomial infections in a Dutch neonatal intensive care unit: Surveillance study with definitions for infection specifically adapted for neonates. J. Hosp. Infect. 61 (4), 300–311. 10.1016/j.jhin.2005.03.014 16221510

