# Supplementary figures and images for: Predicting treatment response to vancomycin using bacterial DNA load as a pharmacodynamic marker in premature and very low birth weight neonates: A population PKPD study
Source: Front Pharmacol. 2023 Feb 16;14:1104482. doi: 10.3389/fphar.2023.1104482 (PMC9978179; doi:10.3389/fphar.2023.1104482)

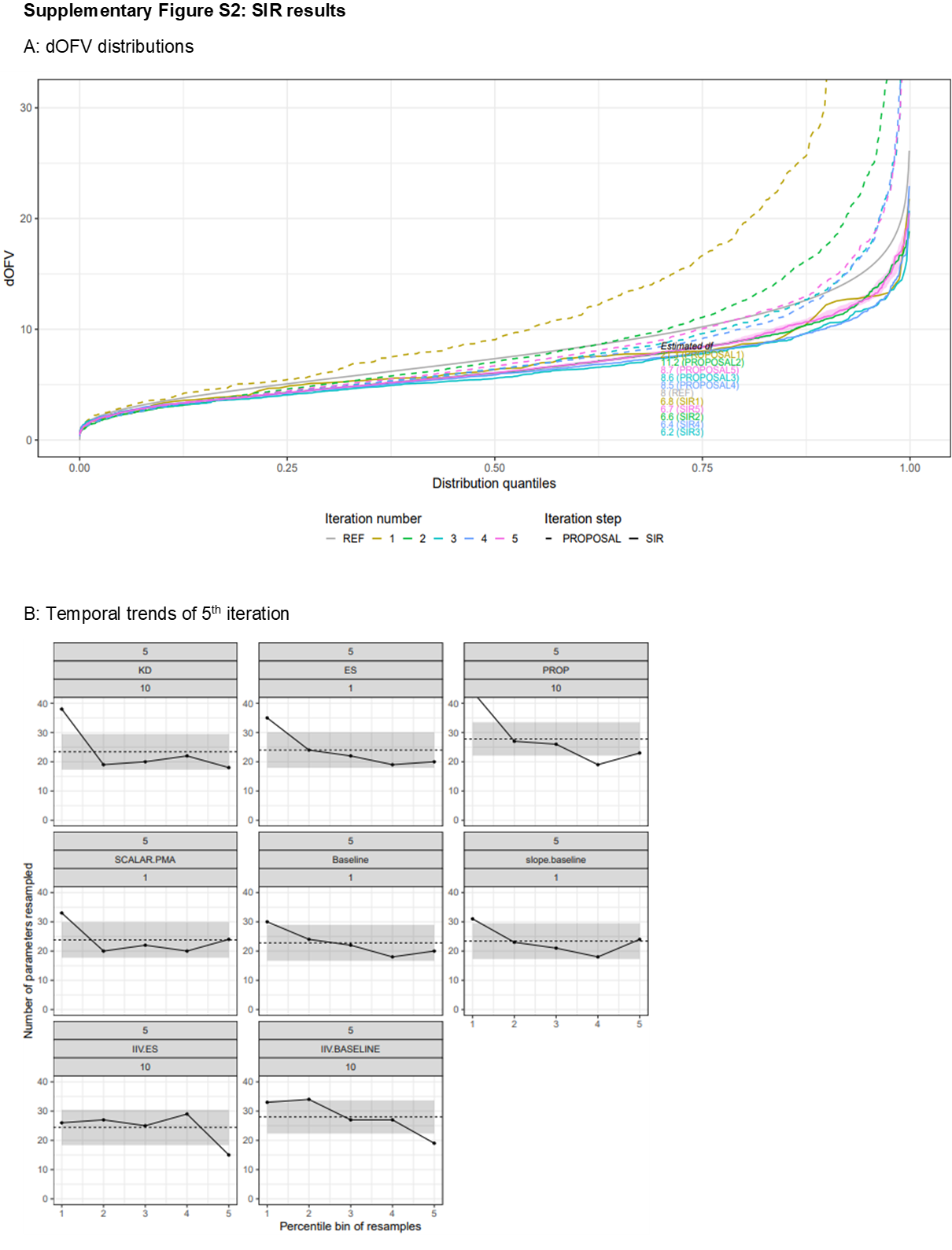

Supplement: Supplementary file 1 [file Image2.TIF]

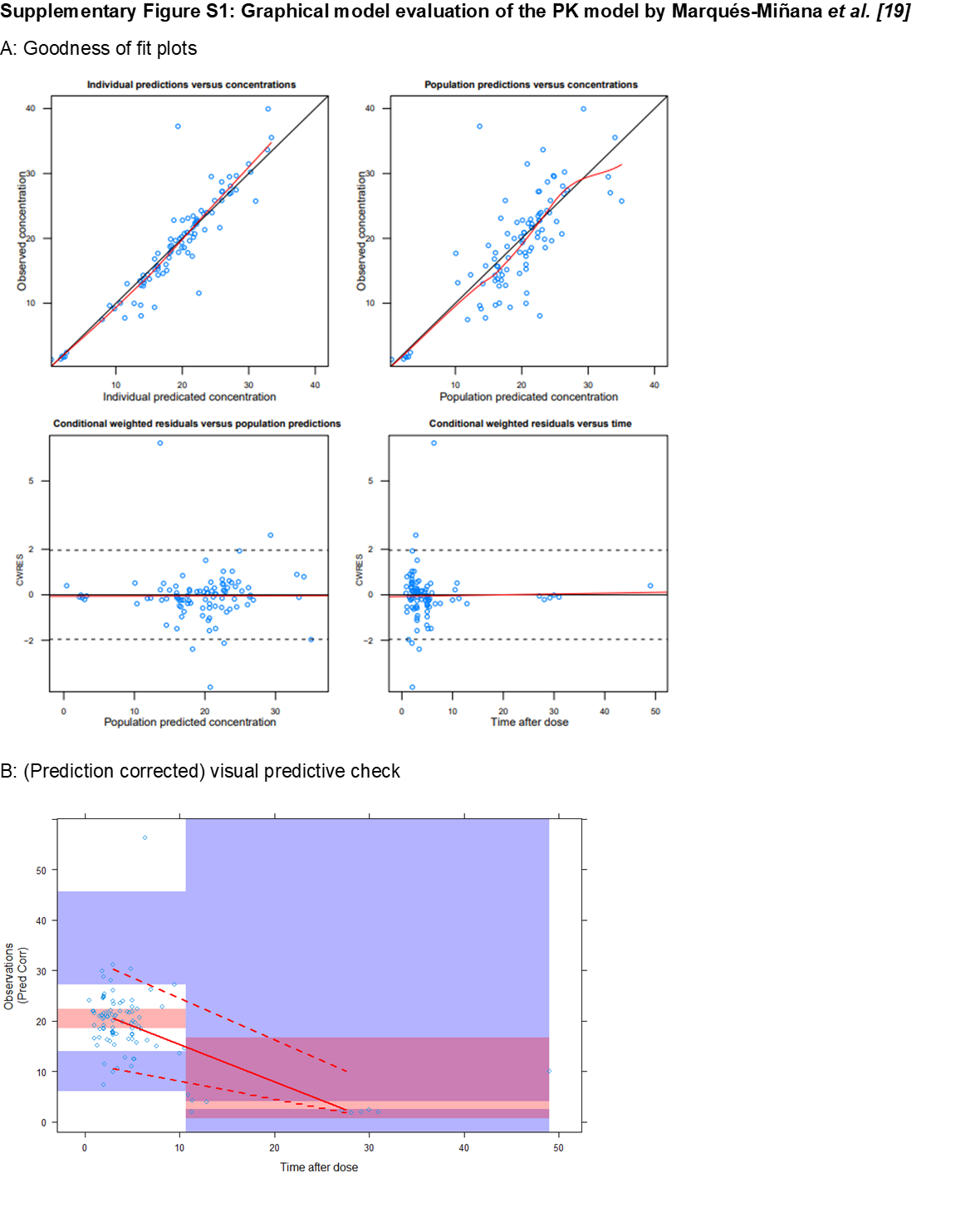

Supplement: Supplementary file 2 [file Image1.TIF]
